# Clinical Characteristics of Newborn Infants Delivered to Pregnant Women With Laboratory-Confirmed COVID-19: A Single-Center Experience From Saudi Arabia

**DOI:** 10.7759/cureus.18573

**Published:** 2021-10-07

**Authors:** Mansour A AlQurashi, Amjed Alattas, Bader Shirah, Ahmad Mustafa, Mohammed Y Al-Hindi, Alyaa Alrefai, Yaser A Faden, Ali Al-Shareef, Eman Al Thuibaiti, Mohammed Hasosah

**Affiliations:** 1 Department of Pediatrics, Neonatology Division, King Abdulaziz Medical City, Ministry of National Guard Health Affairs, Western Region, Jeddah, SAU; 2 College of Medicine, King Saud Bin Abdulaziz University for Health Sciences, Jeddah, SAU; 3 Research Office, King Abdullah International Medical Research Center, Jeddah, SAU; 4 Department of Pediatrics, King Abdullah Medical Complex, King Abdulaziz University, Jeddah, SAU; 5 Department of Obstetrics and Gynecology, King Abdulaziz Medical City, Ministry of National Guard Health Affairs, Western Region, Jeddah, SAU; 6 Department of Emergency Medicine, King Abdulaziz Medical City, Ministry of National Guard Health Affairs, Western Region, Jeddah, SAU; 7 Department of Pediatric Gastroenterology, King Abdulaziz Medical City, Ministry of National Guard Health Affairs, Western Region, Jeddah, SAU

**Keywords:** vertical transmission, saudi arabia, covid-19, coronavirus, breastfeeding

## Abstract

Introduction

In Saudi Arabia and countries around the world, clinical health practice has been transformed by the coronavirus 2019 (COVID-19) pandemic caused by the severe acute respiratory syndrome coronavirus 2 (SARS-CoV-2). During the early days of the pandemic, it was a major challenge to care for pregnant women with laboratory-confirmed COVID-19 and their newborn infants. In this article, we share our experience in the management of newborn infants delivered to mothers with laboratory-confirmed COVID-19.

Methods

A prospective single-center observational study was conducted at King Abdulaziz Medical City in Jeddah, National Guard Health Affairs, Saudi Arabia. Data collection started in March 2020 and was completed in October 2020. The inclusion criteria included mothers with laboratory-confirmed COVID-19 and their newborn infants.

Results

A total of 45 pregnant women with polymerase chain reaction (PCR)-confirmed COVID-19 were included in the study. Their mean age was 30.23±5.92 years. The mode of delivery was spontaneous vaginal delivery in 27 women (60%), cesarean section in 15 women (33.3%), and assisted vaginal delivery in three women (6.7%). Three mothers (6.7%) required intensive care unit admission. A total of 45 babies were born and 25 were females (55.6%), 20 males (44.4%). None of the babies had specific symptoms related to COVID-19. All babies were tested negative on the two COVID-19 nasopharyngeal swabs. Babies were initially admitted to the NICU and one baby required prolonged NICU stay due to extreme prematurity (23 weeks), one baby died due to hypoxic-ischemic encephalopathy and respiratory distress syndrome, and the remaining babies were discharged home in a stable condition.

Conclusion

Our experience suggests that maternal outcomes are generally favorable and no difference between vaginal and cesarean delivery in the risk of virus transmission. With strict implementation of infection prevention measures, mother-to-infant transmission is very unlikely. Early bathing of the newborn infant is preferred to reduce the risk of transmission of infection to newborn infants and the hospital staff. Breastfeeding is safe if performed under strict infection prevention measures.

## Introduction

In Saudi Arabia and countries around the world, clinical health practice has been transformed by the COVID-19 pandemic, which is caused by the SARS-CoV-2 Coronavirus [1]. This disease was declared a global pandemic by the World Health Organization (WHO) on March 11, 2020 [2]. Since then, the virus has spread rapidly and as of July 14, 2021, there were more than 180 million cases around the world, and more than 500,000 confirmed cases in Saudi Arabia [3]. Given the need for strict social isolation, the government implemented at the earlier months of the pandemic a curfew to protect both citizens and expatriates and to limit the spread of this highly infectious virus [4]. King Abdulaziz Medical City-Jeddah, a 751-bed medical complex, was among the healthcare systems in Saudi Arabia to be directly affected by COVID-19. It was prepared to care for a large number of COVID-19 in-patients while simultaneously provide continuous clinical care for patients who require treatment and complex tertiary care. During the early weeks of the pandemic, it was a major challenge to care for pregnant women with laboratory-confirmed COVID-19 and their newborns. This was mainly due to the fear of the effect of this highly contagious disease on the health of the mother and her fetus as well as the potential risk of transmission of the virus from the mother to her newborn and health care workers working in the maternal and neonatal care units. In addition, knowledge of several other aspects was limited in the earlier weeks of the pandemic including the potential vertical transmission of the virus, the safety of breastfeeding, and rooming-in with the mother. In this article, we share our experience in the management of mothers with laboratory-confirmed COVID-19 and their newborn infants.

## Materials and methods

A prospective single-center observational study was conducted at King Abdulaziz Medical City in Jeddah, National Guard Health Affairs, Saudi Arabia. Data collection started in March 2020 and was completed by October 2020. The inclusion criteria included mothers with laboratory-confirmed COVID-19 and their newborn infants. The study period was eight months, and patients whose data are incomplete were excluded. Data were obtained from the patients' files and collected on the data collection sheets.

The protocols at our hospital in terms of caring for pregnant women with polymerase chain reaction (PCR)-confirmed COVID-19 were structured based on the best available evidence at each given time and guided by the guidelines of related scientific bodies in the field of perinatal health. The pregnant woman would be placed in a dedicated negative pressure isolation room prior to the time of delivery, and for vaginal delivery, an isolation room in the labor and delivery suite was dedicated to women with COVID-19, while operative deliveries took place in the main hospital's operating room with dedicated operating room for such cases. All members of the obstetric and neonatal teams were required to wear personal protective equipment for airborne exposure and the women were shifted back to the isolation room until discharge from the hospital.

The management guidelines for infants born to mothers with COVID-19 were followed and started from the delivery room weather in the emergency department or the obstetric labor and delivery ward. The neonatal team would wear personal protective equipment for airborne exposure to receive the baby from the obstetrics colleagues. The neonatal team then moved the infants to the resuscitation room where the rest of the team was waiting and early care/resuscitation was carried out as per the Neonatal Resuscitation Program (NRP) guidelines. Once the baby was stable after initial care, whole-body bathing with soap and water was done before transfer to the neonatal intensive care unit (NICU). Both stable and unstable infants were moved immediately after initial care to a dedicated negative pressure isolation room in the neonatal unit. The baby would be tested for COVID-19 by nasopharyngeal swab at 24 and 48 hours of life. The baby would initially receive formula feeding. Mothers were assisted to initiate breast milk expression after delivery if their condition allows, which was done after proper hand hygiene and while wearing a face mask. The baby was not allowed to room-in with the mother, and if the baby is negative on the two consecutive screening tests and fit for discharge, he/she can be discharged with the appropriate caregiver if the family situation was cleared by the infection prevention and control department. This practice was changed in the last few months wherein newborns were discharged home with their mothers provided all precautionary measures are in place.

Descriptive statistical tests (mean and standard deviation, frequency, percentage) were calculated using the Statistical Package for Social Sciences (SPSS) version 23.0. This study was approved by the Institutional Review Board (IRB) of King Abdullah International Medical Research Center (KAIMRC).

## Results

A total of 45 pregnant women with PCR-confirmed COVID-19 were included in the study. Their mean age was 30.23±5.92 years, and the mean gestational age in weeks was 37.13±4.24 weeks (range 23-41 weeks). The mean length of hospital stay was 7.30±7.25 days (range 2-35 days). Seven women (15.6%) had chest X-ray abnormalities. The majority of the women did not have comorbidities (39 women), while the others had co-morbidities including hypothyroidism, hepatitis B surface antigen positive, systemic lupus erythematosus, and sickle cell trait. Thirty-four women had no obstetric complications, while (5) had gestational diabetes, (2) had premature rupture of membranes, (1) had abruption placenta, (1) had preeclampsia, (1) had cholestasis of pregnancy, and (1) had chorioamnionitis. The mode of delivery was spontaneous vaginal delivery in 27 women (60%), cesarean section in 15 women (33.3%), and assisted vaginal delivery in 3 women (6.7%). Eight women (17.8%) had a premature delivery (before 37 weeks). Only three women (6.7%) required intensive care unit admission (two for obstetric complications and one for COVID-19-related respiratory complications). The majority of women were discharged home after the observation period in a stable condition (42 women). Two mothers required prolonged hospitalization due to an unstable condition for discharge. One of the women had severe COVID-19 infection that resulted in premature delivery at 23 weeks and required extracorporeal membrane oxygenation (ECMO) and unfortunately died from the disease 48 hours after cesarean section. 

A total of 45 babies were born. Twenty-five were females (55.6%) and 20 were males (44.4%). None of the babies had specific symptoms related to COVID-19. All babies were tested negative on the two COVID-19 nasopharyngeal swabs. One baby had an X-ray of respiratory distress syndrome (prematurity related) and another had a pneumothorax, while the remaining babies had a normal chest X-ray. Babies were initially admitted to the NICU and one baby required prolonged NICU stay due to extreme prematurity (23 weeks), one baby died due to hypoxic-ischemic encephalopathy and respiratory distress syndrome, and the remaining babies were discharged home in a stable condition (Table 1).

**Table 1:** Clinical data of mothers in our study with PCR-confirmed COVID-19 (n=45). PCR: polymerase chain reaction.

Variables	Number (range)	%
Mean age	30.23±5.92 years	
Mean gestational age in weeks	37.13±4.24 weeks (23-41 weeks)	
Mean length of hospital stay	7.30±7.25 days (2-35 days)	
Chest X-ray abnormalities	7	15.6
Comorbidities		
Present	6	13.3
Absent	39	86.7
Obstetric complications		
None	34	75.6
Gestational diabetes	5	11.1
Premature rupture of membranes	2	4.4
Abruption placenta	1	2.2
Preeclampsia	1	2.2
Cholestasis of pregnancy	1	2.2
Chorioamnionitis	1	2.2
Mode of delivery		
Spontaneous vaginal delivery	27	60
Cesarean section	15	33.3
Assisted vaginal delivery	3	6.7
Premature delivery (before 37 weeks)	8	17.8
Intensive care unit admission	3	6.7
Prolonged hospitalization	2	4.4
Sex of the baby		
Females	25	55.6
Males	20	44.4

## Discussion

The COVID-19 pandemic caused major trauma to the healthcare systems around the world, and many aspects of medical practice have changed completely as a response to this pandemic. Pregnant women and newborns are affected by the pandemic situation and are among the groups prone to infection with SARS-CoV-2 and therefore, it has been a challenge to continue providing high-quality medical care for pregnant women and their newborn infants. This study summarizes our experience in caring for this group during the unfortunate days of the pandemic. We discuss the challenges and the changes that were guided by the growing body of evidence in the key issues including isolation, bathing, vertical transmission, and breastfeeding.

The initial reports from China demonstrated that the mode of delivery in women with COVID-19 was a cesarean section in almost all women [5,6]. However, other studies from Europe (the 2nd epicenter of the pandemic) reported high rates of vaginal deliveries (40%-70%) [7-10]. There was no difference in terms of infection transmission between the two modes of delivery. In our hospital, the rate of vaginal delivery was high (60%) compared to others and no difference was found in the outcomes between vaginal and cesarean delivery.

Preterm birth was observed in the previous reports in up to 20% of cases [7-9] and this was marginally higher than the usual rate observed in the general population. In our study, the rate of prematurity was close to the previously reported rate in the literature at 17.8%. Our unit baseline prematurity rate ranges anywhere from 15% to 19%.

In this study, three women (6.7%) required critical care and this is comparable to studies from Europe (7.5%-9%). This low number of intensive care unit admission may indicate that COVID-19 has a better and less severe course of the disease compared to H1N1 flu, severe acute respiratory syndrome (SARS), and Middle East respiratory syndrome (MERS) epidemics [11].

Although there is evidence suggesting the disadvantages of isolating newborns early in life and for several reasons [12], we elected to separate all of them initially to avoid potential transmission of the infection to the newly born infants and its subsequent complications. Rooming-in was initially thought to be high-risk for transmission given the speculations that the SARS-CoV-2 may be airborne, and for that, the practice was justified at the earlier period, and women agreed to that to protect their babies. Our practice has changed more recently to allow rooming-in provided that all precautionary measures are implemented, and an individualized case-by-case decision is currently practiced. 

Newborn bathing in the context of SARS-CoV-2 has been reported in the literature with variable practices [12-15]. Some earlier studies encouraged the immediate bathing of newborns in order to reduce the risk of exposure to pathogens for both newborns and hospital staff and therefore reducing the risk of infection spread13-15. However, vaginal secretions and amniotic fluid were tested in some other studies and shown that SARS-CoV-2 is not detected in these specimens [12]. Other studies found no difference in infection transmission between early and delayed bathing (after 24 hours of birth) [12]. In our hospital, we practiced immediate bathing to reduce the risk of transmission of the virus and to protect the hospital staff.

Early reports suggested the possibility of in-utero vertical transmission of SARS-CoV-2 [16-19]. However, there is no enough evidence on how mother-to-infant vertical transmission occurs, and several reports published more recently confirmed that vertical transmission of the virus seems highly unlikely if proper measures were taken during delivery and post-partum [20]. In our study, we did not observe transmission in the study population. This could be attributed to the strict infection control measures implemented and also indicates that with the proper performance of infection prevention methods, transmission is unlikely.

Breastfeeding was a controversial topic at the beginning of the pandemic as earlier reports suggested that the shedding of SARS-CoV-2 RNA in breast milk was observed. Therefore, human breast milk was thought initially a potential route to transmit SARS-CoV-2 from mother to newborn infant during breastfeeding [21]. It remains unknown whether viral RNA represents infectious viral particles. On the contrary, more recent studies demonstrated that breast milk contains IgM and IgA antibodies to SARS-CoV-2 suggesting that breastfeeding might have a “protective role” in neonates against transmission of COVID-19 [22]. In addition, some reports showed that a few newborns tested positive for SARS-CoV-2 after mothers had skin-to-skin contact and breastfed without face masks [23]. Therefore, recommendations changed to consider breastfeeding under strict infection prevention techniques including a face mask for the mother, gloves, and cleaning the breast before skin-to-skin contact [24]. In our hospital, we elected to place all newborn infants on formula feeding in the earlier weeks since the evidence was not conclusive on the safety of breastfeeding or using expressed breast milk, however, with the current evidence demonstrating the safety of breastfeeding under controlled circumstances, we may consider breastfeeding safe to the newborn and our practice had changed since then. Breast milk PCR was not performed for the population of our study.

The psychological impact of the infection prevention methods was also profound on pregnant women who not only fear for themselves from this potentially deadly virus but more for their babies in-utero and post-natally. Moreover, the mothers were affected by the inability to hold their babies, isolation from the baby for a few days, and inability to breastfeed. This issue, although was difficult to handle, was understood by the mothers given the potential risk of transmission of the virus to their newborn infants particularly in the earlier days and its possible complications. Therefore, conducting research to address the important aspects of delivery and postnatal care is important to improve the existing evidence and support the development of clinical practice guidelines that take into consideration the mother's psychological status while still providing the utmost protection for the newborn infant.

## Conclusions

The COVID-19 pandemic has created significant challenges in the care of pregnant women and their newborn infants. Our experience suggests that maternal outcomes are generally favorable and no difference between vaginal and cesarean delivery in the risk of virus transmission. With strict implementation of infection prevention measures, mother-to-infant transmission is very unlikely. Early bathing of the newborn infant is preferred to reduce the risk of transmission of infection to the newborn infant and the hospital staff. Breastfeeding is safe if performed with strict infection prevention measures. Further studies are needed to further expand the growing body of evidence related to maternal and perinatal health.
